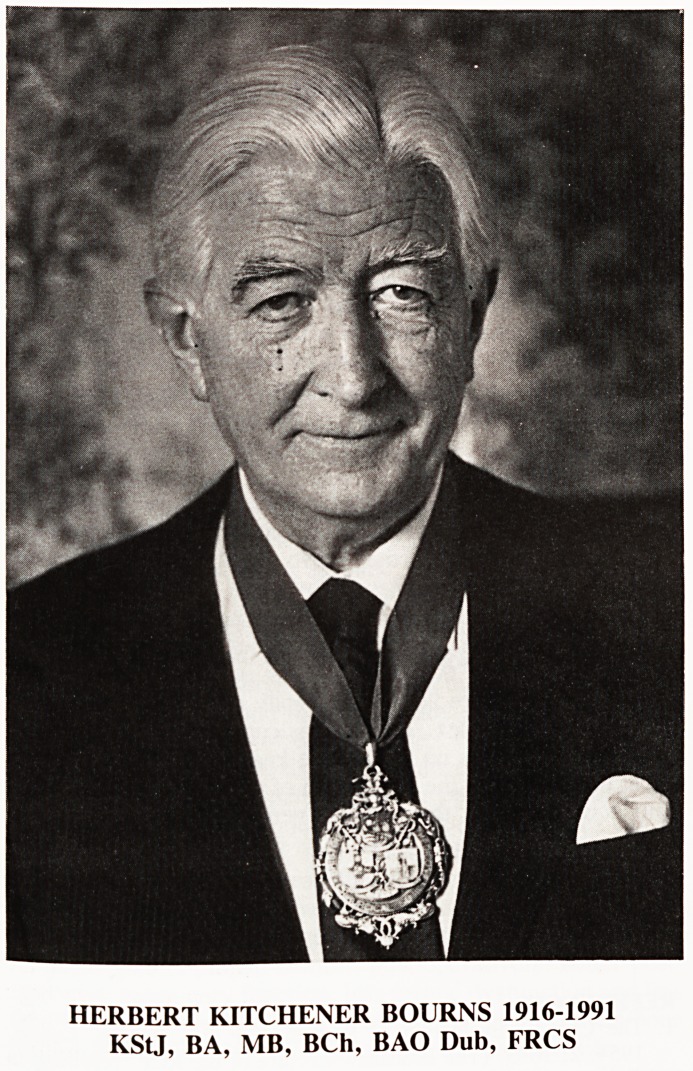# Herbert Kitchener Bourns

**Published:** 1991-12

**Authors:** 


### Obituary

**HERBERT KITCHENER BOURNS 1916-1991**
**KStJ, BA, MB, BCh, BAO Dub, FRCS**

Herbert Bourns who died suddently on October 12th 1991 was born in County Galway in July 1916. He was the son of a Protestant farmer and landowner and the sixth of seven children. His schooling was at St. Columba's College near Dublin and his medical education at Trinity College Dublin where he graduated in 1939. On the outbreak of war he came to England and worked initially at the E.M.S. Hospital in Exminster and later as a house surgeon and RSO at the Royal Devon and Exeter Hospital. In 1942 he joined the RAMC as a trainee Orthopaedic surgeon at Shaftesbury Military Hospital. In due course he became an Orthopaedic Specialist with various Military Hospitals. He landed in Europe on D+4 with a field hospital that was set up at Caen, later he moved to Brussels where he served until VE day. Though still in Europe on VJ day he did not escape far Eastern service and was sent to India, Burma and Malaysia. He was demobilised in January 1947 and became a surgical registrar at the Bristol Royal Infirmary. In 1949 he gained the F.R.C.S. and was appointed Senior Surgical Registrar to Mr. Gordon Paul and Mr. John Pocock. In 1952 he became the first Consultant Surgeon in charge of the Accident and Emergency Department at the Bristol Royal Infirmary with beds at the BRI and at Frenchay/Cossham hospitals.

As Emergency surgeon he came into frequent contact with the Ambulance Services who brought into hospital patients to whom they had given first aid and in many cases whose lives they had saved. He came to admire and respect their work and volunteered his services as a lecturer and examiner to the St. John Ambulance Service. Later he became Director of the St. John's Association in Avon and was recently made a Knight of the Order of St. John of Jerusalem in recognition of his services.

He was an active member of the BMA and was Chairman of the Bristol Division. He was Chairman of the Regional Training Committee, President of the Bristol Medico-Chirurgical Society and the Cossham Medical Society and President of the Surgical Club of South West England.

In the Bristol University Medical School he held the position of Clinical Lecturer, he enjoyed teaching and his gentle and quietly humorous manner was appreciated equally by students and patients.

He was one of the original group involved in the foundation of St. Peter's Hospice in Bristol and was active in its original planning. In the early days the emphasis was upon setting up a domiciliary nursing service. Later as funds were acquired they built St. Peter's Lodge, the Day Centre and the Educational Wing was added. In these efforts his wife Joan contributed equally and their devoted work in the difficult early days was the foundation of its present success in helping so many cancer sufferers live peacefully through the last days of their lives.

He was a committed Christian and a member of the Chistian Medical Fellowship. Active in Freemasonry he was a member of St. Vincent's Lodge, Bristol and a founder member of Gerard Lodge, Keynsham. In his youth a hockey player he remained keenly interested in sport and was President of Bristol United Hospital Rugby Football Club from 1955 until 1970. He enjoyed the country life at his wife's family farm in Devon and was a keen gardener at his home in Bristol.

In spite of a busy professional life he found time for his many other activities and gave himself energetically and selflessly to causes in which he believed. His manner calm and kind but enlivened with his quick wit and dry humour. He was beloved by all who had the good fortune to know him or be his patient.

His private life was ideally happy. He met his wife Joan when he was a resident and she a Red Cross nurse at the Exminster Hospital in 1941, they were married the following year and have four sons and 10 grandchildren.

## Figures and Tables

**Figure f1:**